# The Association between β-Thalassemia Major (β-TM) and Cardiac Complications: Recent Insights

**DOI:** 10.2174/011573403X376074250630065253

**Published:** 2025-08-07

**Authors:** Jalal Taneera, Hussein S. Huwaijah, Reem Qannita, Ayah Alalami, Ayah Dib, Alaa AlHajji, Amani Alhajji, Reem El-Tahrawi, Mohamed A. Saleh, Mahmoud M. Ramadan, Ahmed S. Ibrahim, Firdos Ahmad, Mawieh Hamad

**Affiliations:** 1 College of Medicine, University of Sharjah, Sharjah, United Arab Emirates;; 2 Research Institute for Medical and Health Sciences, University of Sharjah, Sharjah, United Arab Emirates;; 3 Center of Excellence of Precision Medicine, University of Sharjah. Sharjah, United Arab Emirates;; 4 Department of Cardiology, Faculty of Medicine, Mansoura University, Mansoura 35516, Arab Republic of Egypt;; 5 Department of Radiology, Faculty of Medicine, Ain Shams University, Cairo, Arab Republic of Egypt;; 6 College of Health Sciences, University of Sharjah, Sharjah, United Arab Emirates

**Keywords:** β-thalassemia major, cardiac complications, iron overload, cardiomyopathy, biomarkers, iron chelation therapy

## Abstract

β-thalassemia Major (β-TM) is a severe hereditary disorder characterized by insufficient synthesis of β-globin chains, resulting in chronic anemia and lifelong dependence on regular blood transfusions. Despite advancements in therapeutic modalities, cardiac complications, including atrial fibrillation, cardiomyopathy, and pulmonary hypertension, continue to be significant contributors to morbidity and mortality among β-TM patients. These persistent cardiovascular risks underscore the urgent need for early, accurate detection and the implementation of personalized assessment strategies to improve patient outcomes.

The prevalence of cardiac complications is notably high, with studies reporting affected individuals in up to 71% of the β-TM population. This highlights cardiac pathology as a predominant clinical concern in this population. The primary underlying mechanism is iron overload, predominantly resulting from chronic transfusional therapy. Excess iron accumulates in the myocardium, leading to myocardial siderosis, the development of dilated cardiomyopathy, and an increased risk of life-threatening arrhythmias.

Cardiac magnetic resonance imaging (cMR), particularly T2* imaging, remains the gold standard for quantifying myocardial iron deposition and guiding therapeutic interventions. Emerging biomarkers, such as Growth Differentiation Factor-15 (GDF-15) and galectin-3, have shown potential for early detection of cardiac involvement and risk stratification, with the prospect of improving clinical outcomes through timely and targeted interventions. This review aims to discuss the prevalence and pathophysiology of cardiac complications in β-thalassemia major (β-TM), delineate risk factors, including serum ferritin levels, iron chelation therapy, age, genetic predispositions, and splenectomy, and evaluate current diagnostic and monitoring strategies. Furthermore, the utility of novel biomarkers, including follistatin and other emerging candidates, for early detection and prognosis is discussed, highlighting their potential to facilitate personalized management approaches that may reduce cardiac morbidity and mortality. In conclusion, integrating advanced imaging modalities such as cMR, novel biomarker profiling, and individualized risk stratification, considering ferritin levels, genetic factors, and splenectomy status, may significantly enhance early detection and intervention strategies, ultimately mitigating the burden of cardiac complications in β-TM.

## 1 INTRODUCTION

β-Thalassemia Major (β-TM) is an autosomal recessive hematologic disorder resulting from mutations in the β-globin gene, which lead to defective hemoglobin synthesis, chronic anemia, and lifelong dependence on blood transfusion therapy [1, 2]. Clinically, it is classified into three categories: minor (asymptomatic carrier state), intermediate (moderate anemia), and major (β-thalassemia major), which is characterized by transfusion dependence [3, 4]. β-TM represents the most severe phenotype, necessitating regular transfusions to alleviate hypoxia and suppress marrow hyperactivity [2]. However, chronic transfusions contribute to systemic iron overload, predominantly accumulating in the myocardium, liver, and endocrine organs, thereby precipitating multi-organ dysfunction [5]. Despite advancements in iron chelation therapy, cardiac complications, including cardiomyopathy, arrhythmias, and pulmonary hypertension, remain the primary causes of morbidity and mortality in β-TM, accounting for up to 71% of deaths in untreated cohorts [6].

Cardiac pathology in β-TM arises from persistent anemia, iron-mediated toxicity, and secondary metabolic disturbances [7]. Dilated cardiomyopathy, observed in approximately 18-25% of transfusion-dependent patients, results from iron deposition within cardiomyocytes *via* dysregulated calcium channels, namely L-type and T-type, disrupting excitation-contraction coupling and promoting oxidative stress, mitochondrial dysfunction, and ferroptosis [8-10]. Early-stage cardiac involvement often present as diastolic dysfunction (prevalence ~59%) or restrictive physiology, progressing to systolic heart failure as iron overload intensifies (notably with cardiac T2* MRI values <20 ms) [11, 12]. Arrhythmias, particularly atrial fibrillation (~40% in patients over 40 years of age), are associated with iron-induced atrial fibrosis and suppression of CaV1.3-dependent calcium currents. In contrast, ventricular tachyarrhythmias and sudden cardiac death correlate with heterogeneous ventricular repolarization secondary to focal iron deposits [13]. Pulmonary hypertension, though mechanistically less well-defined, may develop secondary to chronic high-output states or vascular remodeling, with reported prevalence ranging from 8% to 30% [14, 15]. Additionally, autoimmune myocarditis can contribute to heart failure independently of iron burden, highlighting the multifactorial nature of cardiac injury in this population [6]. Current management strategies include intensified iron chelation, utilizing agents such as deferoxamine and deferiprone to reduce myocardial iron loading, complemented by pharmacologic therapy for arrhythmias, including β-blockers and amiodarone [16]. Emerging biomarkers and advanced cardiac MRI techniques (*e.g*., T1 and T2 mapping) facilitate the early detection of subclinical myocardial changes. Multidisciplinary care and routine monitoring remain crucial for mitigating cardiac morbidity. This review aims to delineate the prevalence and associated risk factors of cardiac complications in β-thalassemia major (β-TM), and to examine contemporary assessment methodologies and the prospective utility of early predictive biomarkers for improved prognosis.

## 2 METHODOLOGY

A comprehensive literature search was conducted across PubMed/MEDLINE, Scopus, Web of Science, and Google Scholar from January 2024 to April 2025. Search terms included “β-thalassemia major,” “cardiac complications,” “cardiomyopathy,” “atrial fibrillation,” “pulmonary hypertension,” “iron overload,” “myocardial iron,” “biomarkers,” “risk factors,” “iron chelation therapy,” and “cardiac MRI,” using various combinations and MeSH terms. The inclusion criteria encompassed peer-reviewed original research, systematic reviews, and meta-analyses focusing on β-TM and associated cardiac pathology in both pediatric and adult human populations. Exclusion criteria included studies on other forms of thalassemia (*e.g*., α-thalassemia), animal or *in vitro* studies, case reports, editorials, and non-peer-reviewed articles.

### 2.1 Prevalence of Cardiac Complications in β-TM

Cardiac complications are the foremost cause of mortality in patients with β-TM [6, 17]. Repeated transfusional iron loading results in myocardial iron accumulation, disturbing cellular homeostasis, and precipitating cardiomyopathy. The prevalence of iron overload-induced cardiomyopathy is markedly higher in β-TM compared to the general population [18]. Current estimates indicate that up to 71% of β-TM patients develop cardiac complications, regardless of their transfusional dependency [3]. Major cardiac manifestations include atrial fibrillation, cardiomyopathy, and myocardial infarction. Atrial fibrillation is observed in approximately 33% of β-TM patients [14], with some reports indicating incidence rates as high as 40%, particularly among older cohorts [19]. In a cohort study of 60 thalassemia patients (30 with β-thalassemia major and 30 with intermedia), β-thalassemia major patients demonstrated increased susceptibility to arrhythmias, such as sinus tachycardia, supraventricular tachycardia, and right bundle branch block [20].

The prevalence of dilated cardiomyopathy varies across studies. One investigation involving patients receiving regular transfusions and iron chelation reported that 37% exhibited severe cardiomyopathy with impaired contractile function, while 31% displayed mild dysfunction [21]. Another study of 52 patients with severe β-TM and heart failure documented a high prevalence (83%) of left ventricular dilated cardiomyopathy, with an additional 17% presenting with right ventricular failure [22]. A third study, which involved 110 patients with thalassemia intermedia, revealed that 66 patients (around 60%) exhibited right ventricular dilation and heart failure [23].

Myocarditis, an inflammatory condition of the myocardium, appears less frequently but remains clinically relevant. Its prevalence varies with age, disease severity, and comorbidities. One extensive cohort study reported an acute myocarditis prevalence of approximately 5% in β-TM patients, with an average age of presentation at 5 years [18]. Patients with myocarditis frequently develop dilated cardiomyopathy and exhibit a significant reduction in left ventricular ejection fraction [6]. In severe β-TM cases, acute infectious myocarditis can lead to chronic left-sided heart failure in about 28% of patients, with an average age of 3.5 years, compared to matched controls. Overall, the literature underscores a substantial burden of cardiovascular complications in β-TM patients, posing significant clinical management challenges.

## 3 RISK FACTORS ASSOCIATED WITH CARDIAC COMPLICATIONS IN Β-TM

While traditional cardiovascular risk factors, such as diabetes mellitus, dyslipidemia, hypertension, age, smoking, sedentary lifestyle, and genetic predispositions, contribute to overall disease burden [24], iron overload remains the predominant driver of cardiac pathology in β-TM [6]. Preclinical studies suggest that divalent metal transporter 1 (DMT1) and L-type calcium channels (LTCC) play a role in myocardial iron influx [25-27]. Under physiological conditions, iron uptake occurs *via* transferrin receptor-mediated endocytosis; however, in β-TM, excess non-transferrin-bound iron (NTBI) accumulates, with ferrous iron (Fe^2+^) exploiting LTCC pathways due to their promiscuity for divalent cations. Fe^2+^ competes with Ca^2+^ entry, disrupting calcium homeostasis, impairing excitation-contraction coupling, and promoting oxidative stress, mitochondrial dysfunction, and ferroptosis [8-10]. Iron catalyzes Fenton reactions, generating hydroxyl radicals, which damage lipids, proteins, and DNA, destabilize mitochondrial membranes, reduce ATP production, and trigger apoptosis *via* caspase-3 activation [28]. Excess myocardial iron shortens the action potential duration, thereby increasing the arrhythmogenic potential. Chronic inflammation and immunogenetic factors further exacerbate oxidative stress and calcium dysregulation, leading to systolic and diastolic dysfunction. Paradoxically, low serum ferritin levels have also been associated with cardiac complications, indicating a nuanced relationship between iron biomarkers and cardiotoxicity [29, 30].

**Fig. (1):**
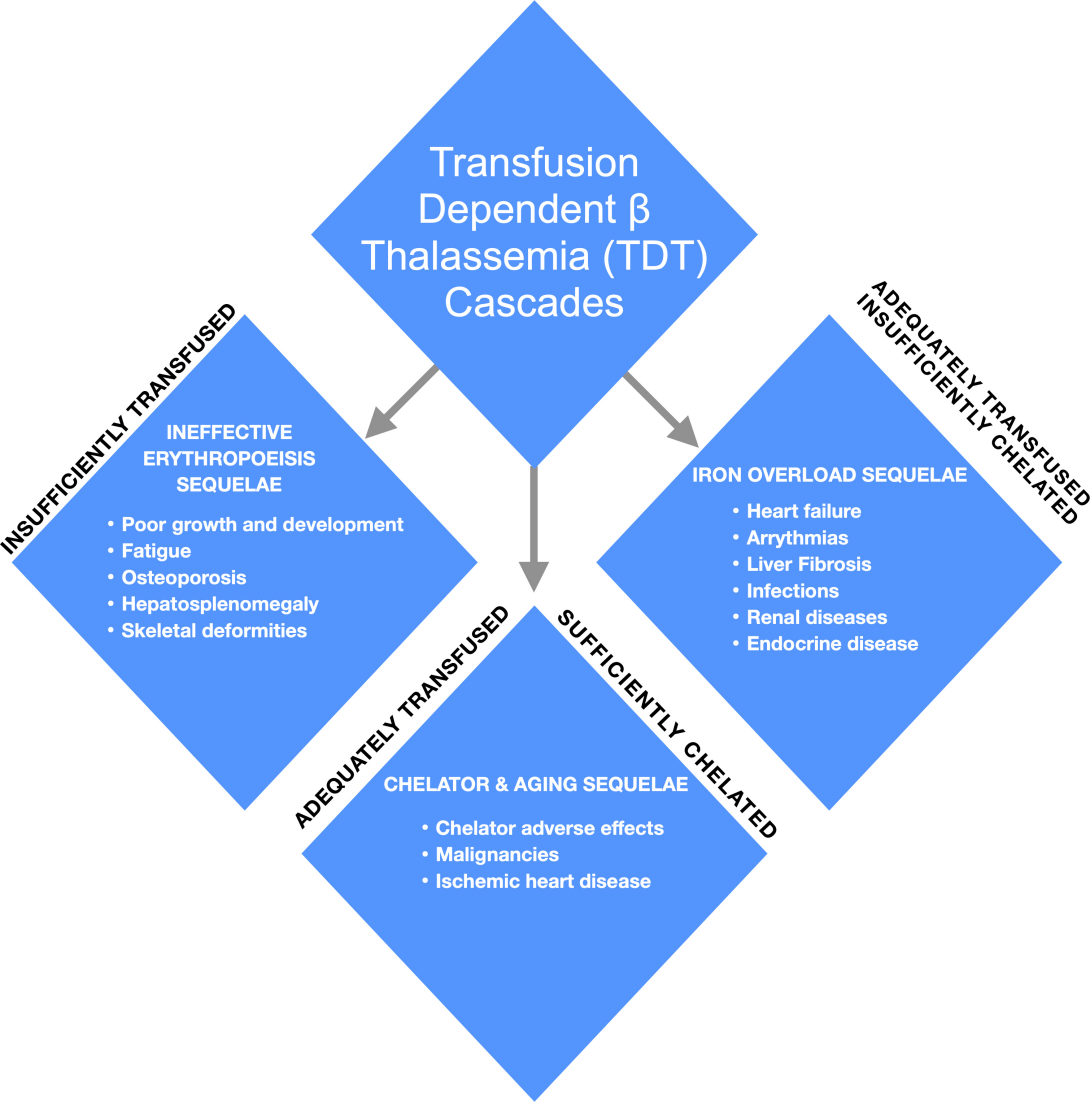
The general clinical profile of transfusion-dependent β-thalassemia (TDT). Hb: hemoglobin, IE: ineffective erythropoiesis.

In non-transfusion-dependent thalassemia (NTDT), increased gastrointestinal iron absorption primarily drives systemic overload, whereas in transfusion-dependent thalassemia (TDT), iron accrues mainly through transfusional iron loading, compounded by inadequate excretion mechanisms [3, 31, 32]. The risk of cardiac complications in transfusion-dependent thalassemia (TDT) varies with the adequacy of transfusional and chelation therapy. Inadequate transfusions can promote extramedullary hematopoiesis and leukocyte-mediated arterial inflammation [31-33], while suboptimal iron chelation exacerbates myocardial iron loading, increasing the risk of cardiomyopathy and arrhythmias [31]. Estimates indicate that iron-induced cardiomyopathy develops in 25-36.7% of TDT patients, with prevalence influenced by adherence to chelation therapy and monitoring strategies (Fig. **1**) [34].

Age is a significant risk factor for cardiac complications, particularly in TDT populations [35, 36]. Over time, ongoing myocardial iron deposition contributes to cardiomyopathy and heart failure. Age-related changes in pharmacokinetics and pharmacodynamics may influence therapeutic responses, further complicating management [37]. Studies have shown that the absence of iron overload-related cardiomyopathies is observed in patients under 15 years of age in certain populations, such as Emirati patients [38], with similar findings reported in North American cohorts [39].

Genetic factors, including HLA genotype, apolipoprotein E polymorphisms, and β-globin genotype, influence the severity of cardiac complications. For example, β-globin genotype severity correlates with myocardial iron load and left ventricular function; individuals homozygous for β° alleles exhibit higher susceptibility to cardiomyopathy [40-42]. The GSTM1 null genotype, which impairs antioxidant defenses, has also been linked to increased hepatic and myocardial iron loading, although evidence remains conflicting [43-45].

Splenectomy introduces additional risks, including thrombotic events and pulmonary hypertension, by impairing the clearance of circulating cellular debris and promoting endothelial injury *via* free hemoglobin and pro-vasoconstrictive mediators [46]. The removal of the spleen exacerbates vascular injury, leading to pulmonary vasoconstriction, intimal hyperplasia, and right ventricular strain, especially in patients with ongoing hemolysis. Pulmonary hypertension affects approximately 25-54% of patients with splenectomy, with some studies reporting up to 54% exhibiting microvascular alterations suggestive of thromboembolic phenomena [47-50]. The pathophysiology involves platelet activation, abnormal erythrocyte aggregation, and increased adhesion of nucleated red blood cells, further elevating pulmonary vascular resistance [13, 47].

## 4 ASSESSMENT STRATEGIES FOR CARDIAC COMPLICATIONS IN Β-TM PATIENTS

Effective management of cardiac complications is paramount, given that they constitute the leading cause of mortality among individuals with β-TM [32]. Recent advances, including the development of iron-chelating agents and the refinement of techniques for monitoring iron levels, have significantly improved life expectancy, particularly among patients with transfusion-dependent thalassemia (TDT). For instance, previous work has demonstrated that patients with serum ferritin levels below 2,500 μg/L have an estimated cardiac disease-free survival rate of approximately 91% after 15 years, compared to a markedly lower survival rate in those with serum ferritin exceeding this threshold [51]. Nonetheless, as patients age, the prevalence of additional comorbidities and health complications continues to rise [5]. This highlights the crucial need for effective management strategies, combined with ongoing education and awareness initiatives, to mitigate the effects of cardiac pathologies in this population.

**Fig. (2):**
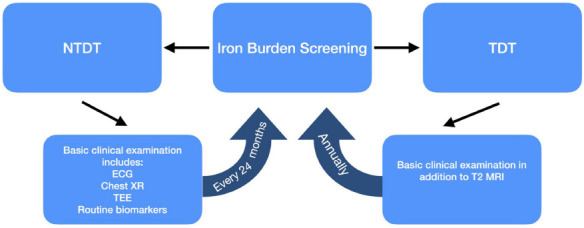
Clinical cardiac evaluation in patients with β-TM.

Routine cardiac evaluation, particularly in asymptomatic individuals, is crucial for the early detection of functional impairments [52]. Although TDT and NTDT-thalassemia share some pathogenic mechanisms, their management approaches differ substantially (Fig. **2**) [53].

**Fig. (3):**
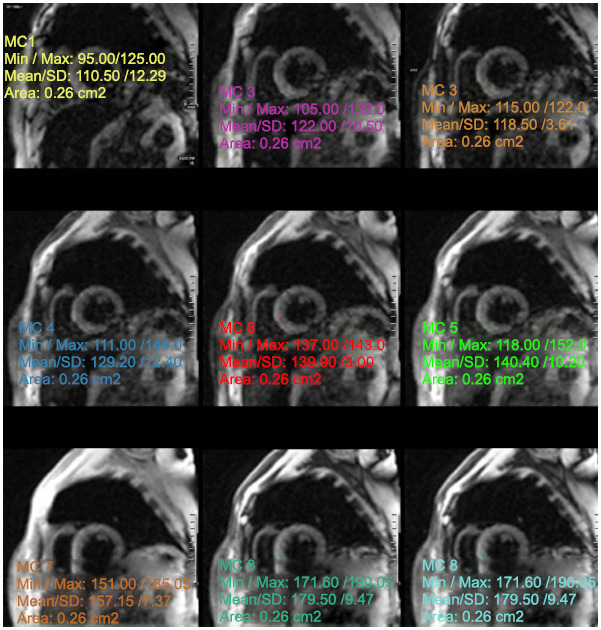
A dedicated ECG-gated cardiac MRI utilizing various progressive TE (Time to Echo) values demonstrating no evidence of myocardial iron overload, with a calculated T2* value of 32.8 ms (normal is above 20 ms). Notably, the liver iron concentration (LIC) was elevated at 6.25 mg/g (normal is below 2 mg/g), with progressively increased darkening of the liver observed across successive TEs, indicating hepatic iron deposition.

Cardiac magnetic resonance imaging (cMRI), utilizing T2* techniques, is a cornerstone in assessing myocardial iron deposition and structural heart changes. Myocardial T2* measurement provides a reliable quantitative indicator of myocardial iron overload, critical for guiding therapeutic interventions aimed at preventing or treating ventricular dysfunction [54]. Standard myocardial T2* values are defined as greater than 20 ms. Values between 10 and 20 ms indicate mild to moderate iron loading, warranting close monitoring and potential therapeutic adjustments (Fig. **3**).

**Fig. (4):**
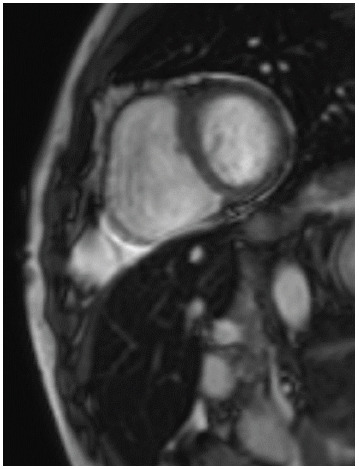
A dedicated ECG-gated cardiac MRI utilizing various progressive TE values demonstrating mild myocardial iron overload, with a calculated T2* value of 17.2 ms (normal is above 20 ms). Notably, the liver iron concentration (LIC) was elevated at 17.5 mg/g (normal is below 2 mg/g), indicating severe hepatic iron overload in this case. Low signal intensity and darkening of the liver and cardiac parenchyma are observed, consistent with iron deposition.

Severe iron overload is indicated by T2* values of 10 ms or less [55], with values below 10 ms being associated with a 160-fold increased risk of heart failure within the subsequent 12 months [56]. Rose *et al.* confirmed that international validation efforts have reinforced the recognition and endorsement of T2* monitoring as a vital tool in assessing myocardial iron burden [26]. Currently, T2* measurement is recommended for inclusion in the annual surveillance protocol for individuals at risk of cardiac siderosis due to repeated transfusions. All patients with β-TM should undergo yearly evaluations, including physical examination, chest radiography, cardiac echocardiography, and resting ECG. The frequency of these assessments may be adjusted based on individual risk factors, with more frequent follow-ups advised for those with underlying cardiac conditions (Fig. **4**).

For β-thalassemia major (β-TM) patients with sustained iron overload, iron chelation therapy is essential for effective management. Cardiac complications related to iron overload can be significantly prevented using iron chelators such as deferasirox (DFX), deferiprone (DFP), and deferoxamine (DFO), to maintain cardiac magnetic resonance (cMR) T2* values above 20 ms [57]. Even mild impairment in ventricular function warrants intensified iron chelation, emphasizing the importance of early intervention [57]. Combination therapy with oral deferiprone and parenteral deferoxamine has proven effective in clearing cardiac iron deposits. A fundamental strategy to prevent iron-related cardiac complications involves maintaining a cMR T2* value greater than 20 ms and a pre-transfusion hemoglobin level of approximately 10 g/dL [58].

## 5 POTENTIAL BIOMARKERS

Patients with β-TM often experience multiple comorbidities, including chronic hemolytic anemia, iron overload, and ineffective erythropoiesis, all contributing to cardiac complications. Identifying reliable biomarkers associated with these complications is vital for early diagnosis, risk stratification, and monitoring disease progression. These biomarkers provide valuable insights into the cardiovascular pathophysiology in β-TM (see Table **1**).

Serum ferritin is the most widely used marker for assessing total body iron stores; however, it may not accurately reflect myocardial iron load [59]. Elevated ferritin levels above 2500 μg/L suggest an increased risk of cardiac complications and warrant further evaluation [60]. However, low ferritin levels do not necessarily indicate a reduced risk of heart failure. Notably, single cross-sectional ferritin measurements are insufficient for reliably assessing long-term myocardial iron status, which can lead to misleading conclusions.

Apolipoproteins A1 (Apo-A1) and B (Apo-B) are key components of lipid metabolism. Apo-B promotes atherogenesis, while Apo-A1 exhibits anti-atherogenic effects [61]. Patients with β-TM often exhibit lower levels of both Apo-A1 and Apo-B, as well as elevated triglycerides, compared to healthy controls [62, 63]. Oxidative stress markers such as platelet activation biomarkers (PAB) are frequently elevated in β-TM, indicating increased oxidative damage and its role in cardiovascular complications [64].

Growth differentiation factor 15 (GDF-15) and carotid intima-media thickness (CIMT) are emerging as valuable biomarkers for assessing atherosclerosis risk. Elevated GDF-15 levels, along with increased CIMT, have been observed in β-TM patients, correlating with clinical and hematological parameters, and suggesting their utility in predicting atherosclerosis [65]. Similarly, increased plasma GDF-15 levels are associated with a higher risk of cardiovascular disease [66]. Elevated galectin-3 levels serve as indicators of myocardial fibrosis due to iron overload and are predictive of heart failure [67]. Increased levels of natriuretic peptides (NP) are strongly linked with higher mortality and morbidity in heart failure, especially in patients with reduced ejection fraction (HFrEF), providing prognostic information and guiding treatment [68].

In asymptomatic children with β-thalassemia major (β-TM), GDF-15, galectin-3, and NP levels do not reliably predict myocardial iron accumulation; however, a weak association exists between serum galectin-3 and hepatic iron deposition [69]. Markers of oxidative stress, such as malondialdehyde (MDA) and protein carbonyl groups, are elevated in both TDT and NTDT, reflecting ongoing oxidative damage that can contribute to cardiovascular pathology [70-72]. Inflammatory markers, such as C-reactive protein (CRP) and interleukin-6 (IL-6), are associated with cardiac dysfunction in β-TM. However, CRP levels do not correlate directly with other biochemical risk factors, indicating a complex interplay between inflammation and cardiac risk [73-75].

Hemolytic markers, such as serum lactate dehydrogenase (LDH), can predict cardiac complications; however, further validation is needed. Homocysteine levels, which positively correlate with lipid risk factors like triglycerides and very low-density lipoprotein (VLDL), reflecting the multifactorial nature of cardiovascular risk [77, 78], tend to be lower in β-TM patients compared to healthy individuals. Follistatin (FST), a circulating protein primarily secreted by the liver, has been associated with several metabolic disorders. Elevated FST levels can precede the onset of type 2 diabetes by up to 20 years and are independently linked to increased mortality, heart failure, and stroke risk [79-82]. Trimethylamine N-oxide (TMAO), derived from microbial metabolism of choline, carnitine, and betaine, is associated with inflammation and cardiometabolic risk factors, including increased risk of atherosclerosis and cardiovascular events [83-85]. Elevated TMAO levels correlate with markers such as CRP, lipoproteins, and blood pressure, emphasizing its role in cardiovascular risk stratification.

## CONCLUSION, REMARKS, AND FUTURE PERSPECTIVES

The high prevalence of cardiac complications in β-TM highlights the critical importance of early detection and effective management strategies to improve patient outcomes. Further research is necessary to elucidate the complex interactions among iron overload, oxidative stress, and genetic predisposition. Advances in imaging modalities remain essential for monitoring myocardial iron deposition and guiding therapeutic decisions. While current treatments, such as iron chelation, have demonstrated promise, challenges persist in optimizing regimens to reduce cardiac risks. Future efforts should focus on identifying novel, reliable biomarkers to enable early diagnosis, risk stratification, and tailored interventions that can significantly enhance clinical management. Additionally, developing personalized medicine approaches that incorporate individual genetic, biochemical, and clinical profiles could transform the management of β-TM, potentially reducing adverse outcomes. Implementing routine cardiac evaluation, preventive measures, and increasing awareness among healthcare providers are vital steps toward improving the quality of life for these patients. As our understanding of β-TM advances, collaborative research across disciplines will be crucial in addressing ongoing challenges.

## Figures and Tables

**Table 1:** Potential serum biomarkers for cardiac complications in β-TM patients.

**S. No.**	**Serum Biomarkers**	**Clinical Indication**
1.	Ferritin	Direct indicator for body iron overload
2.	NT	Mortality and morbidity in heart failure patients with reduced ejection fraction
3.	GDF-15	Correlated with the development of atherosclerosis and increased cardiovascular risk
4.	IL6	Elevated linked with cardiac dysfunction
5.	CRP	Oxidative markers and inflammatory biomarkers
6.	MDA	Indicators of oxidative stress and cardiovascular damage
7.	Galectin-3	An indicator of myocardial fibrosis and heart failure
8.	TMNO	Correlated with cardiovascular mortality or major adverse cardiovascular events
9.	FST	Elevated follistatin levels are associated with an increased risk of mortality and cardiometabolic disorders

## LIST OF ABBREVIATIONS

CIMT: Carotid Intima-Media Thickness
cMR: Cardiac Magnetic Resonance Imaging
CRP: C-Reactive Protein
DMT1: Divalent Metal Transporter 1
GDF-15: Growth Differentiation Factor-15
LDH: Lactate Dehydrogenase
LTCC: L-Type Calcium Channels
MDA: Malondialdehyde
NTBI: Non-Transferrin-Bound Iron
NTDT: Non-Transfusion-Dependent Thalassemia
TDT: Transfusion-Dependent Thalassemia
TMAO: Trimethylamine N-oxide
VLDL: Very Low-Density Lipoprotein
β-TM): β-Thalassemia Major
